# Antioxidants Hydroxytyrosol and Thioredoxin-Mimetic Peptide CB3 Protect Irradiated Normal Tissue Cells

**DOI:** 10.3390/antiox13080961

**Published:** 2024-08-07

**Authors:** Katrin Borrmann, Fabian Martin Troschel, Kathrin Annemarie Brücksken, Nancy Adriana Espinoza-Sánchez, Maryam Rezaei, Kai Moritz Eder, Björn Kemper, Hans Theodor Eich, Burkhard Greve

**Affiliations:** 1 Department of Radiation Oncology, University Hospital Münster, 48149 Münster, Germany; 2 Department of Gynecology and Obstetrics, University Hospital Münster, 48149 Münster, Germany; 3 Institute of Physiological Chemistry and Pathobiochemistry, University of Münster, 48149 Münster, Germany; 4 Biomedical Technology Center, Medical Faculty, University of Münster, 48149 Münster, Germanybkemper@uni-muenster.de (B.K.)

**Keywords:** antioxidants, oxidative stress, ionizing radiation, inflammation, migration, skin, vascularization

## Abstract

Reducing side effects in non-cancerous tissue is a key aim of modern radiotherapy. Here, we assessed whether the use of the antioxidants hydroxytyrosol (HT) and thioredoxin-mimetic peptide CB3 (TMP) attenuated radiation-induced normal tissue toxicity in vitro. We used primary human umbilical vein endothelial cells (HUVECs) and human epidermal keratinocytes (HaCaT) as normal tissue models. Cells were treated with HT and TMP 24 h or immediately prior to irradiation. Reactive oxygen species (ROS) were assessed via luminescent- and fluorescence-based assays, migration was investigated using digital holographic microscopy, and clonogenic survival was quantified by colony formation assays. Angiogenesis and wound healing were evaluated via time-dependent microscopy. Secreted cytokines were validated in quantitative polymerase chain reaction (qPCR) studies. Treatment with HT or TMP was well tolerated by cells. The application of either antioxidant before irradiation resulted in reduced ROS formation and a distinct decrease in cytokines compared to similarly irradiated, but otherwise untreated, controls. Antioxidant treatment also increased post-radiogenic migration and angiogenesis while accelerating wound healing. HT or TMP treatment immediately before radiotherapy increased clonogenic survival after radiotherapy, while treatment 24 h before radiotherapy enhanced baseline proliferation. Both antioxidants may decrease radiation-induced normal tissue toxicity and deserve further pre-clinical investigation.

## 1. Introduction

Radiation is a key paradigm in cancer therapy as more than half of cancer patients are recommended radiotherapy as part of their treatment [1]. Modern radiotherapy has reduced the incidence and severity of therapy-related side effects given precise target volume delineation and highly conformal, intensity-modulated radiation dose application. However, adverse reactions to treatment attenuate quality of life and remain a major obstacle to intensified therapy and, ultimately, improved cancer cure [2]. Thus, reducing side effects remains a major priority in radiation treatment [3].

Skin reactions are among the most frequent radiation-induced adverse reactions as external beam radiation therapy, the most frequent radiotherapy application, requires radiation to penetrate the skin to reach the tumor [4]. In breast cancer, 90% of irradiated patients have early local skin reactions and chronic late effects such as fibrosis/scleroderma, telangiectasia, atrophy, skin necrosis, and secondary tumors [5,6]. The skin is sensitive to ionising radiation (IR) due to its rapid proliferation rate, and the endothelial cells of small blood vessels are irreversibly damaged by IR, leading to long-term damage [7].

The mechanisms behind these radiation-induced side effects are closely linked to oxidative stress [8]. Radiation-induced ionizations result in free radicals, for instance, reactive oxygen species (ROS) and reactive nitrogen species (RNS). Examples of the most important ROS that are produced in tissues after irradiation are superoxide anions, hydrogen peroxide, hydroxyl radicals, singlet oxygen, and peroxyl radicals. These radicals induce DNA damage and a pro-inflammatory cascade of cytokines and chemokines. Effects may persist beyond the radiation treatment period, curtailing tissue regeneration in the long term [4,9]. Wound healing is a complex process involving inflammation, cell proliferation and tissue remodeling that requires the dynamic and coordinated activity of biochemical, physiological, and cellular processes [10,11,12]. Endogenous antioxidant systems such as superoxide dismutase (SOD), glutathione peroxidase (GPX) and aldehyde dehydrogenase (ALDH) can neutralize free radicals, but their capacity is limited [13]. Therefore, we hypothesized that the use of antioxidants could have a positive effect on wound-healing processes and reduce radiation-induced skin reactions [14].

In this experimental study, the effects of using the antioxidants hydroxytyrosol (HT) and the thioredoxin-mimetic peptide CB3 (TMP) as radioprotective agents are investigated. The central aspect of this work is the selective administration and localized effect of the antioxidants. The topical application directly to the skin over the healthy tissue is intended to create a protective barrier that reduces oxidative damage caused by reactive oxygen species (ROS) generated during radiotherapy. This targeted application ensures that the antioxidants act exclusively in healthy tissue and do not reach the tumor.

**Figure 1:**
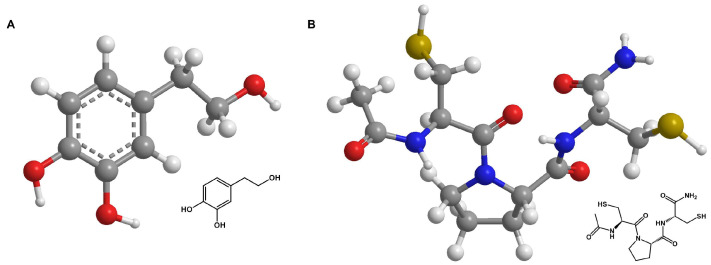
Skeletal formulars of antioxidants. Skeletal formulars of HT (**A**) and TMP (**B**). Carbon atoms are shown in grey, oxygen atoms in red, nitrogen atoms in blue, sulfur atoms in yellow, and hydrogen atoms in white. The dashed line represents the conjugated double bonds in the benzene ring (**A**).

The water-soluble phenylethanoid hydroxytyrosol (HT) (Figure 1A) and its derivatives are commonly found in olives and olive oil [15]. This natural component has been reported to be antioxidative, anti-inflammatory, antimicrobial, and antidiabetic, as well as cardio- and neuroprotective [16]. Recent studies have shown HT to have positive effects on proliferation and migration in HaCaT cells through MMP 9 activation and promote angiogenesis in HUVECs [17,18]. It also inhibits the TNFα-induced nuclear translocation of NFκB in porcine vascular endothelial cells [19]. HT has a phenolic structure with an ortho-dihydroxy group. This structure is particularly effective in scavenging free radicals, as the hydroxyl groups can donate hydrogen atoms to reduce ROS to less reactive molecules.

The TrxR/Try system, consisting of thioredoxin reductase, thioredoxin, and nicotinamide adenine dinucleotide phosphate (NADPH), is one of the crucial redox systems in cells and plays an important role in maintaining the balance of oxidative stress in cells [20]. This led to the development of thiol-based, cysteine-containing amides, called thioredoxin mimetic (TXM) peptides, which should mimic Trx1 activity and increase the redox state of cells [21,22,23,24]. TMP, the substance investigated here (Figure 1B), was identified as part of this group [20]. The antioxidative effect originates from the proteolysis of TMP into multiple cysteine-containing molecules that form a redox cluster. The thiol groups of the cysteines in the peptide react with ROS and also reduce them to less reactive molecules. Each fragment in the cluster functions as a ROS scavenger, which makes TMP and other members of the TXM family highly antioxidative [20]. Several studies reported that treatment with TMP caused lower ROS levels and the decrease in or inhibition of mitogen-activated protein kinase (MAPK) activity in vitro [22,25,26] and in vivo [23,27] for various pathologies (e.g., oxidative stress related disorders, diabetes mellitus, mild traumatic brain injury, cardiovascular and allergic airway disease). A subsequent decrease in caspase-3 mediated a reduction in cell apoptosis. Hemling et al. showed that treatment with TMP resulted in lower ROS and improved VEGFR-2 receptor levels in primary human fetoplacental endothelial cells (HEPCs) and that the cells were more responsive to VEGF-A, which stimulates angiogenesis [26]. Both antioxidants have shown promising results for various oxidative stress-related diseases in previous studies. However, the use of both antioxidants to protect against radiation-induced skin reactions during or after radiotherapy has been barely or not at all studied.

Both antioxidants were assessed in primary human umbilical vein endothelial cells (HUVECs) and human epidermal keratinocyte (HaCaT) cell cultures to reflect different compartments and functionalities of the skin. We aimed to understand whether treatment with antioxidants alleviated radiation-induced pro-apoptotic, antiproliferative, and pro-inflammatory effects, thus supporting post-radiation normal tissue wound healing.

## 2. Materials and Methods

### 2.1. Chemicals

Hydroxytyrosol (HT) was kindly provided by Prof. Dr. Lothar Heinrich (Institute for Biochemistry, University of Münster, Münster, Germany) and can be purchased from Sigma-Aldrich (Cat.-No. H4291, St. Louis, MO, USA). Thioredoxin-mimetic peptide CB3 (TMP) was custom-made and kindly provided by Dr. Rinesh Godfrey (Molecular Cardiology, University Hospital Münster, Münster, Germany). The molecular structural formulae were created with ChemDraw Ver. 22.2 (Revvity Signals Software Inc., Waltham, MA, USA).

The concentrations were selected based on the best available data and practical limitations. The decision to use a concentration of 100 µM was based on the best results from MTT tests on HUVECs (see Section 3.1) and in agreement with other studies using this concentration [28,29,30]. This concentration allowed the study of the proliferative effects of HT in the optimal range without reaching the potential anti-angiogenic effects observed at higher concentrations [31]. Therefore, it is recommended in the literature to use concentrations not higher than 100 µM for studies on endothelial cells [32]. In selecting the TMP concentration, we were guided by the literature data for in vitro studies. Cohen-Kutner et al. performed experiments with 100 µM TMP [25]. Canesi et al. used concentrations ranging from 0.001 to 100 µM [27], while Atlas used concentrations between 50 and 100 µM [20]. Bachnoff et al. obtained the best results with a treatment of 100 µM compared to other concentrations [22].

### 2.2. Cell Lines, Cultivation and Treatment

Human epidermal keratinocytes (HaCaT) (ATCC, Manassas, VA, USA) were cultured in DMEM containing 10% fetal calf serum (FCS), 1% Penicillin/Streptomycin solution (both PAN Biotech, Aidenbach, Germany), and 25 mM HEPES buffer (Roth, Karlsruhe, Germany).

Primary human umbilical vein endothelial cells (HUVECs) (PromoCell, Heidelberg, Germany) were cultured up to passage four in Endothelial Cell Growth Medium 2 (ECGM-2, PromoCell, Heidelberg, Germany) and supplemented with the provided supplement mix (PromoCell, Heidelberg, Germany) according to the manufacturer’s instructions. Then, 1% Penicillin/Streptomycin solution (PAN Biotech, Aidenbach, Germany) was added. All cells were grown in a humidified atmosphere of 5% CO_2_ at 37 °C. If not otherwise specified in the following experimental sections, the cells were treated with 100 µM HT or 100 µM TMP in Opti MEM I (HaCaT; GIBCO, Burlington, ON, Canada) or ECGM-2 (HUVECs; PromoCell, Heidelberg, Germany) either 24 h before or during irradiation. Given the antioxidant potential of DMEM [33] and the high FCS content in the normal DMEM medium, Opti MEM I was used for the antioxidant treatment of HaCaT cells. In order to homogenize the growth conditions between the cell lines, we explicitly wanted to avoid any interfering FCS-induced growth signals. After irradiation, the media were replaced with standard culture media.

### 2.3. Irradiation

Irradiation was performed using a TrueBeam linear accelerator (Varian, Palo Alto, CA, USA). Gantry was fully opened to a field size of 40 × 40 cm. The table was positioned at a distance of 1 m from the gantry. Six-well plates with cells were positioned within the lightfield of the gantry. A previously described and validated setup was used for irradiation [34]. The dose rate was 4.8 Gy per minute. In total, 6 MV photons were generated and a filter was used to achieve flatness. Doses of 2, 4, and 6 Gy were applied to the cells to represent relevant clinical doses.

### 2.4. MTT Assay

The MTT (3-(4,5-dimethylthiazol-2-yl)-2,5-diphenyltetrazolium bromide) assay was used to establish the appropriate concentration of HT and TMP and determine the metabolic activity and, hence, indirectly, the viability of HaCaT cells and HUVECs after antioxidant treatment. Concentrations of 0.1, 1, 10, and 100 µM were used for both antioxidants. The cells were incubated for 24 h with the respective antioxidant concentrations. Subsequently, cells were washed with PBS and stained with MTT solution (Sigma-Aldrich, St. Louis, MO, USA) according to the manufacturer’s instructions. Colorimetric density was measured using a TriStar LB942 ELISA reader (Berthold Technologies, Bad Wildbad, Germany) at 570 and 650 nm, as previously described [35]. The measurements were normalized to untreated controls.

### 2.5. ROS Assays

The level of reactive oxygen species (ROS) was detected directly after irradiation using the fluorogenic probe CellROX Green (Life Technologies, Darmstadt, Germany). HaCaT cells were seeded with a density of 0.15 × 10^6^ cells per well into a 12-well plate containing round glass coverslips. For the HUVEC collagen, I-coated 8-well chambers (Nunc Lab-Tek Chamber Slide System, Permanox plastic, Thermo Fisher Scientific, Waltham, MA, USA) were used (0.01 × 10^6^ cells/well). After 24 h, the incubation medium was removed, and cells were treated with HT- or TMP-containing media, as described above. For a positive control, 100 µM Menadion (Sigma-Aldrich, St. Louis, MO, USA) was added to cells for 1 h to induce oxidative stress, whereas unstained cells were used as a negative control/blank. CellROX Green was added to each well at a final concentration of 5 µM shortly before irradiation with a dose of 2 Gy.

After irradiation, cells were incubated for 30 min at 37 °C before fixation with 3.7% paraformaldehyde (SERVA Electrophoresis GmbH, Heidelberg, Germany) in 1× PBS. Nuclei were stained with Hoechst 33342 (Sigma-Aldrich, St. Louis, MO, USA) for 30 min at room temperature. Cells were then visualized by fluorescence microscopy (10×, Axiophot, Zeiss, Jena, Germany), and fluorescence intensity was measured as the mean grey value with ImageJ Ver. 1.53 (NIH, Bethesda, MD, USA). The results were normalized against an untreated and unirradiated baseline control.

### 2.6. Flow Cytometry Analysis of γH2AX

HaCaT cells and HUVECs were treated with antioxidants, as described, and irradiated with 2 Gy. Cells were harvested and fixed with 70% ethanol immediately and 2 h after irradiation. For staining, the cells were permeabilized with 0.25% Triton X 100 and blocked with 5% FCS. The cells were then stained with the primary antibody against anti-phospho-histone-H2AX (1:500, Merck Millipore, Burlington, MA, USA). The goat anti-mouse antibody AlexaFluor488 (1:500, Invitrogen, Waltham, MA, USA) was used as a secondary antibody. Cells were also stained with propidium iodide and analyzed by flow cytometry, as described before [36,37,38,39,40,41] (CyFlow Space, Partec, Münster, Germany), using a blue laser at 488 nm and a red laser at 635 nm. Unirradiated controls were used to set the thresholds for determining the percentage of γH2AX-positive cells using FloMax software V. 2.8. (Quantum Analysis, Münster, Germany).

### 2.7. Cytokine Array Analysis

HaCaT cells and HUVECs were plated at a density of 0.8 × 10^6^ cells per well (6-well plate), respectively, and incubated for 24 h before being treated as described. Then, 48 h after irradiation at 2 Gy, supernatants were collected and centrifuged (1500 rpm, 10 min). Aliquots were stored at −20 °C until analysis by RayBio Human Cytokine Antibody Array 5 (RayBiotech, Inc. Norcross, GA, USA) according to the manufacturer’s instructions. Chemiluminescent signals were detected with a FUSION SL (Vilber Lourmat, Marne-la-Vallée Cedex, France) device and quantified with ImageJ Ver. 1.53 (NIH, Bethesda, MD, USA) using the plugin Protein Array Analyzer developed by Gilles Carpentier [42]. The average intensity was normalized to the positive control.

### 2.8. RNA Isolation, Reverse Transcription and qPCR

For qPCR analysis, the total RNAs of HaCaT cells and HUVECs were isolated 24 h after irradiation using the RNeasy^®^ Mini Kit (Qiagen, Venlo, The Netherlands). For reverse transcription, the High Capacity cDNA Reverse Transcription Kit (Applied Biosystems, Thermo Fisher Scientific, Waltham, MA, USA) was used. Both were utilized as per the manufacturers’ protocols.

The expression levels of CXCL1, IL-6, IL-8, TIMP1, and TIMP2 were determined using TaqMan^®^ Gene Expression Master Mix (Applied Biosystems, Thermo Fisher Scientific, Waltham, MA, USA), with 18S functioning as an endogenous control. All qPCRs were carried out in triplicate for each experiment on a Rotor-Gene Q machine (Qiagen, Venlo, The Netherlands) and analyzed for the relative intensity of fluorescent dye to determine the cycle thresholds. The results were evaluated using the comparative cycle threshold method (2^−∆∆Ct^) and expressed as a fold change [43]. Detailed information about the used TaqMan probes can be found in Supplementary Table S1.

### 2.9. Colony Formation Assay

Colony formation assays were performed to determine the clonogenic capacity and survival of the cells after treatment. HaCaT cells and HUVECs were irradiated with doses of 2, 4, and 6 Gy 24 h or immediately after treatment with antioxidants. Directly after irradiation, cells were harvested and counted as previously described [44]. A defined number of cells were then seeded in triplicate into 6-well plates and incubated for 12 days with the continuous exchange of standard media every two to three days. Grown colonies were then fixed and stained in one step using the Loeffler’s staining solution for 30 min (Supplementary Table S2). After counting colonies of more than 25 cells [45] microscopically (10×, Olympus CKX41 microscope; Olympus, Tokyo, Japan), the plating efficiency (1) and surviving fraction (2) were calculated as follows:

PE = amount of colonies/number of seeded cells,(1)

SF = PE (irradiated cells)/PE (control).(2)

### 2.10. Angiogenesis Assay

HUVECs were seeded into 6-well plates and treated with HT and TMP, as described above. After irradiation with a dose of 2 Gy, the cells were harvested and resuspended in Endothelial Cell Growth Medium 1 (ECGM-1, PromoCell, Heidelberg, Germany) supplemented with the provided supplement mix and 1% Penicillin/Streptomycin solution (PAN Biotech). After determining the cell number, 0.04 × 10^6^ cells were added onto a growth factor-reduced polymerized Matrigel (Corning Inc., Corning, NY, USA) layer and incubated at 37 °C for 3 h. Pictures of 5 independent wells were taken for each sample and analyzed with the Angiogenesis Analyzer Plugin for ImageJ developed by Gilles Carpentier et al. [46]. The obtained results were calculated from three independent experiments and displayed as linked networks; untreated cells were used as the control.

### 2.11. Wound-Healing Assay

HaCaT cells were seeded in a silicone culture-insert (ibidi culture-insert 2 well, ibidi GmbH, Martinsried, Germany) at a density of 0.25 × 10^6^ cells in 70 µL per well and incubated for 24 h. Cells were treated with antioxidants, as described above, and after irradiation with 2 Gy, the inserts were removed to achieve a reproducible cell-free gap of 500 µm. The cells were washed with 1× PBS before adding a standard medium. Pictures of the gaps were taken immediately after removing the inserts and every 24 h afterwards. For each sample, three pictures at different gap locations were taken and analyzed with ImageJ Ver. 1.53. The wound area was calculated as follows:

gap t(*x* h)/gap t(0 h) × 100%. (3)

### 2.12. Migration Measurement via Digital Holographic Microscopy

HaCaT cells were seeded into a black 24-well µ-plate with a clear bottom (ibidi GmbH, Martinsried, Germany) at a density of 0.08 × 10^6^ cells per well. After treatment with antioxidants, as described above, cells were irradiated with 2 Gy and incubated for 1 h at 37 °C. Cells were then washed with 1× PBS, and a standard culture medium was added. Subsequently, digital holographic microscopy (DHM) time-lapse measurements with the setup previously reported by Haiduk et al. [35] were performed to investigate the time-dependent migration and proliferation (dry mass increment) of HaCaT cells after treatment with antioxidants and irradiation. DHM images were taken every 15 min over a period of 24 h, and cell motility was evaluated by the quantification of migration speed and distance with the custom-built software TrackHack (developed by the Center for Biomedical Optics and Photonics and the Biomedical Technology Center, University of Münster, Münster, Germany), as described in previously published studies [47,48].

### 2.13. Statistical Analysis

All in vitro experiments were performed independently at least three times (except Cytokine Array, *n* = 1). Data are shown as mean values with the standard error of the mean (s.e.m.). Significances were calculated using Student’s *t*-test. *p* values ≤ 0.05 were considered significant. Graphs were created and statistical analyses were performed with Prism V. 8 (GraphPad Software, San Diego, CA, USA).

## 3. Results

In this experimental work, the effects of the antioxidants HT and TMP as radioprotective substances were investigated in two cell types representing different layers and functional elements of the skin. The results of this pre-clinical study point to a possible reduction in radiation-induced normal tissue toxicity, indicating that antioxidants may support post-radiogenic wound healing and tissue regeneration.

### 3.1. HT and TMP-Induced Changes in Cell Viability

An MTT assay was performed to determine the optimal concentrations of HT and TMP and ascertain that no toxic effect was exerted on the cells.

**Figure 2:**
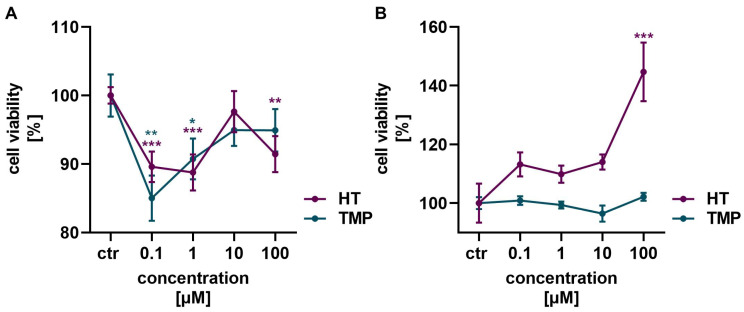
Antioxidants modulate cell viability. Treatment with HT and TMP initially led to a slight reduction in the viability of HaCaT (**A**) at low concentrations, but viability increased again at higher concentrations. An increase in viability was observed for HUVECs (**B**), especially for 100 µM HT. Values represent the mean of three independent experiments with error bars indicating the standard error of the mean (s.e.m.). *p* values < 0.05 were deemed significant (* *p* < 0.05; ** *p* < 0.01; *** *p* < 0.001) and represent the significant difference to the control.

Neither HT nor TMP showed a toxic effect on HaCaT cells and HUVECs in the concentrations of 0.1 to 100 µM used (Figure 2). In HaCaT cells, a slight reduction in cell vitality was observed after the administration of HT and TMP, but viability increased again with higher concentrations of both antioxidants (Figure 2A). HT in particular had a highly positive effect on cell vitality in HUVECs with increasing concentrations (Figure 2B). A concentration of 100 µM was, therefore, used for both HT and TMP in the following experiments.

### 3.2. HT and TMP Decrease ROS Level and DNA Double Strand Breaks after Irradiation

Ionizing radiation induces cell death mostly via the formation of some highly reactive radicals such as ROS, which lead to oxidative stress and may induce irreparable DNA damage.

**Figure 3:**
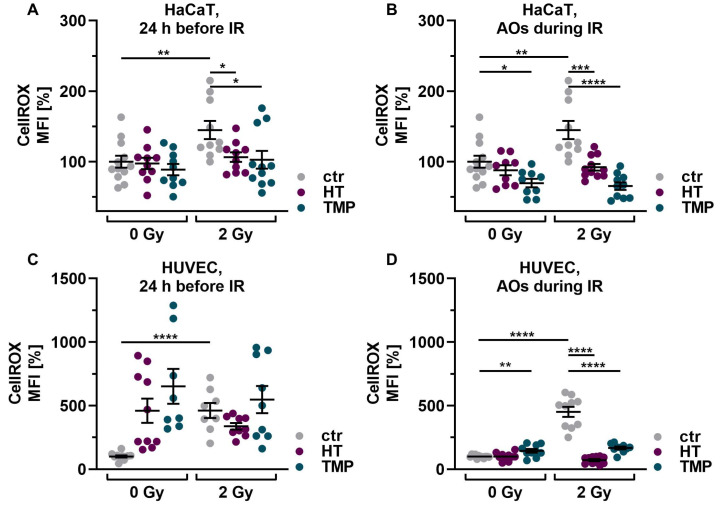

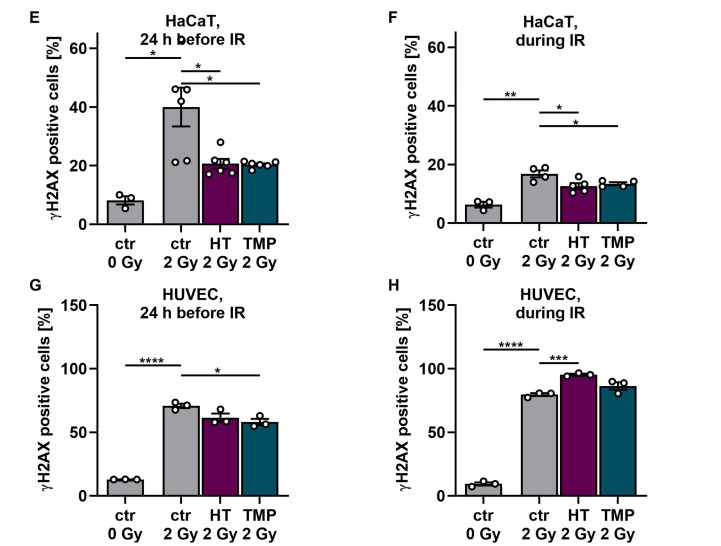
Antioxidant supplementation reduces radiation-induced ROS levels and DNA double-strand breaks in HaCaT cells and HUVEC cultures. Irradiation leads to significantly elevated ROS levels in HaCaT cells (**A**,**B**) and HUVECs (**C**,**D**). Incubation with HT or TMP, respectively, 24 h before (**A**,**C**) or during radiation treatment (**B**,**D**) decreases the amount of radiation-induced ROS significantly. ROS levels in HaCaT cells and HUVECs treated with HT and TMP during irradiation (**B**,**D**) remained almost at the level of unirradiated, treated cells. After irradiation, the number of DNA double-strand breaks increased significantly in otherwise untreated cell cultures (**E**–**H**). Treatment with HT and TMP before irradiation reduced DNA damage in both HaCaT cells (**E**) and HUVECs (**G**), while treatment during irradiation only decreased the number of DNA double-strand breaks in HaCaT cells (**F**) but not in HUVECs (**H**). HUVECs were more susceptible to irradiation-induced double-strand breaks than HaCaT cells. Values represent the mean of three independent experiments, with error bars indicating the standard error of the mean (s.e.m.). *p* values < 0.05 were deemed significant (* *p* < 0.05; ** *p* < 0.01; *** *p* < 0.001, and **** *p* < 0.0001).

First, we investigated the basic effect of HT and TMP on ROS levels in HaCaT cells and HUVECs after irradiation with 2 Gy. Irradiation significantly increased ROS in both cell types in untreated, irradiated cells (ctr 2 Gy) compared to untreated, non-irradiated control cells (ctr 0 Gy). Treatment with 100 µM HT or TMP 24 h before and during irradiation led to a significant reduction in ROS (Figure 3A–D). We also observed that the ROS levels of HaCaT cells and HUVECs treated with HT and TMP during irradiation (Figure 3B,D) remained almost at the level of the unirradiated, treated cells.

Subsequently, we conducted tests on γH2AX to determine the number of DNA double-strand breaks on HaCaT cells and HUVECs after irradiation with 2 Gy and the impact of the two antioxidants on DNA damage (Figure 3E–H). After irradiation, the number of DNA double-strand breaks increased significantly in both untreated cell types. Treatment with HT and TMP 24 h before irradiation led to a reduction in DNA damage in both cell types (Figure 3E–G). However, in the case of treatment of HUVECs with HT and TMP during irradiation, we observed an increase in DNA double-strand breaks compared to untreated irradiated cells (Figure 3H). In addition, we noticed that HUVECs (Figure 3G,H) reacted much more sensitively to irradiation than HaCaT cells (Figure 3E,F), as the increase in DNA double-strand breaks was more pronounced.

### 3.3. Expression and Release of Pro-Inflammatory Cytokines

**Figure 4:**
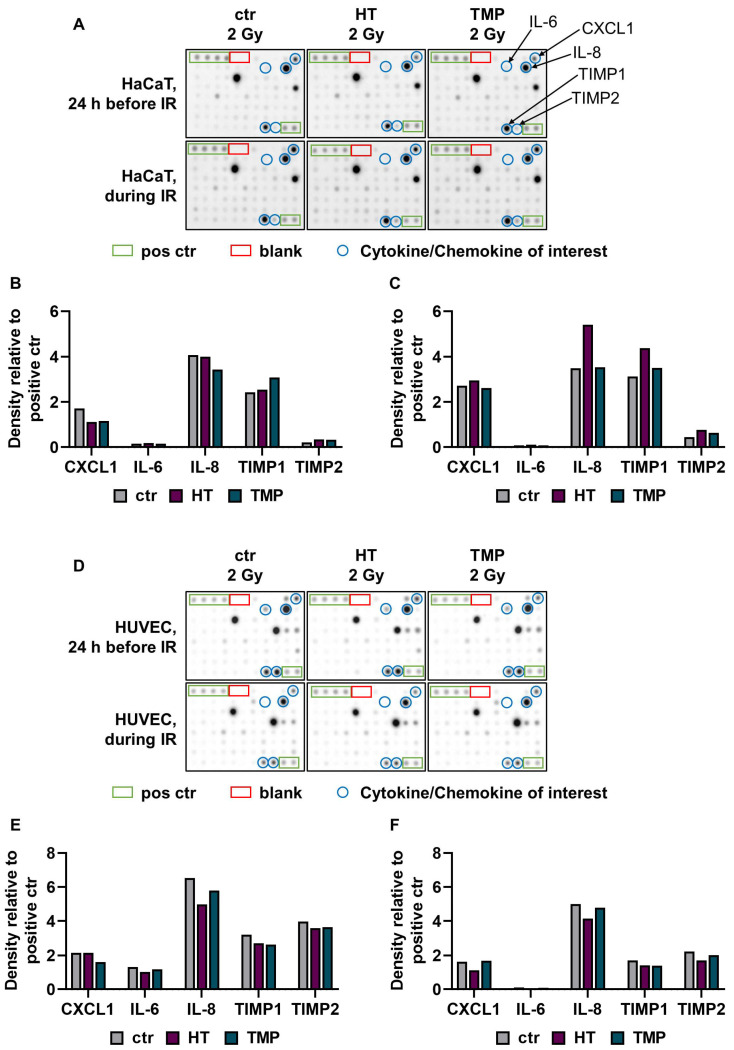
A cytokine array was used to quantify radiation-induced release of pro-inflammatory cytokines. A Cytokine array was performed with supernatants from HaCaT cells (**A**–**C**) and HUVECs (**D**–**F**) treated with HT or TMP 24 h before (**B**,**E**) and during (**C**,**F**) irradiation with 2 Gy (*n* = 1). The supernatants were collected 48 h after irradiation. Chemiluminescence was used to measure and quantify cytokine binding to the membrane. Intensity was evaluated with the help of ImageJ software. Eighty different cytokines were tested, and cytokines with relevant changes are shown in (**B**,**C**,**E**,**F**).

Previous studies indicate that HUVECs release pro-inflammatory cytokines after irradiation, impairing angiogenesis and wound healing [49]. We subsequently performed a cytokine array to investigate whether the use of antioxidants also altered pro-inflammatory signaling in HaCaT cell and HUVEC cultures after irradiation (Figure 4).

The treatment of HaCaT cells with HT and TMP 24 h before irradiation led to a reduction in C-X-C motif chemokine ligand 1 (CXCL1) and an increase in the proteinases TIMP metallopeptidase inhibitor 1 and TIMP metallopeptidase inhibitor 2 (TIMP1 and TIMP2). In addition, TMP caused a reduction in the pro-inflammatory cytokine interleukin 8 (IL-8) (Figure 4B). The incubation of the cells with HT and TMP during irradiation resulted in increases in the levels of CXCL1, IL-8, TIMP1, and TIMP2 (Figure 4C).

For HT-treated HUVECs (24 h before and during irradiation), we observed reductions in IL-6 and IL-8, as well as in TIMP1 and TIMP2 (Figure 4D). Treatment with TMP 24 h before irradiation resulted in lower expressions of CXCL1, IL-6, IL-8, TIMP1, and TIMP2 (Figure 4E), while the treatment during irradiation caused slight reductions in IL-8, TIMP1, and TIMP2 (Figure 4F). IL-6 was barely detectable in HUVECs treated with HT or TMP during irradiation. It must be noted here that the results were generated from one replicate. The results for HUVECs after treatment with HT and TMP but without radiation exposure can be found in Supplementary Figure S1.

**Figure 5:**
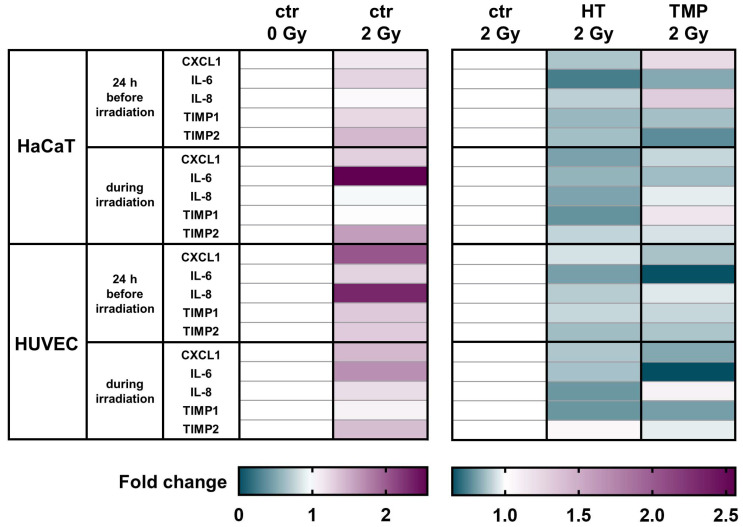
Antioxidants modulate the mRNA levels of different pro-inflammatory cytokines in HaCaT cells and HUVEC cultures. A heatmap of gene expression changes shown by the qRT-PCR of pro-inflammatory cytokines for HaCaT cells and HUVECs after treatment with HT and TMP and irradiation. All experiments were repeated at least three times in triplicate.

To test corresponding intracellular RNA expression changes, we performed qRT-PCR assays. Here, we largely saw the same trends in HaCaT cells and in HUVECs, as inflammatory markers showed a strong expression after irradiation that was significantly attenuated after treatment with antioxidants (Figure 5).

CXCL1 levels were significantly elevated in untreated HaCaT cells and HUVECs after irradiation. A treatment with HT or TMP led to the downregulation of CXCL1 in HaCaT cells and HUVECs in both treatment regimens.

We observed a significant upregulation of IL-6 after irradiation for both cell types. HT and TMP treatment caused decreases i IL-6 in HaCaT cells and in HUVECs treated 24 h before and during irradiation.

No changes in IL-8 levels were detected in untreated HaCaT cells after irradiation, whereas a treatment with TMP 24 h before irradiation caused an upregulation. In HUVECs, IL-8 was significantly upregulated after irradiation, and treatment with HT or TMP led to a downregulation.

TIMP1 was also upregulated in untreated HaCaT cells and HUVECs upon irradiation. HT treatment reduced TIMP1 levels in HaCaT cells and HUVEC cultures in both treatment regiments. TMP treatment 24 h before irradiation led to decreased TIMP1 levels in both cell types, whereas treatment with TMP during irradiation resulted in a slight upregulation in HaCaT cells and a downregulation in HUVECs.

We also found that TIMP2 was significantly upregulated after irradiation in untreated HaCaT cells and HUVECs and that treatment with HT or TMP 24 h before and during irradiation led to a reduction in TIMP2. Table 1 displays a summary of all qPCR results, including s.e.m and significance levels. Fold changes in untreated cells after irradiation related to control 0 Gy can be found in Supplementary Table S3.

### 3.4. Treatment with HT and TMP during Irradiation Boosts Radioresistance in HaCaT Cells and HUVECs

**Figure 6:**
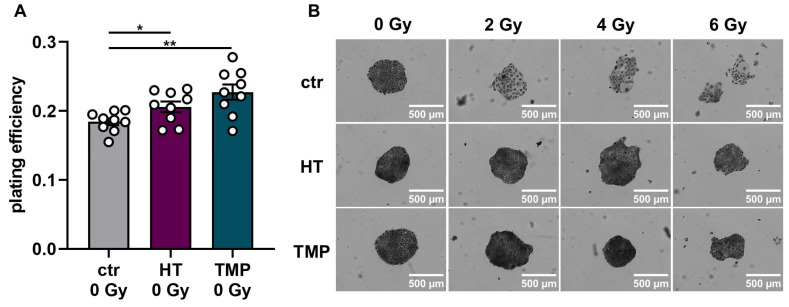

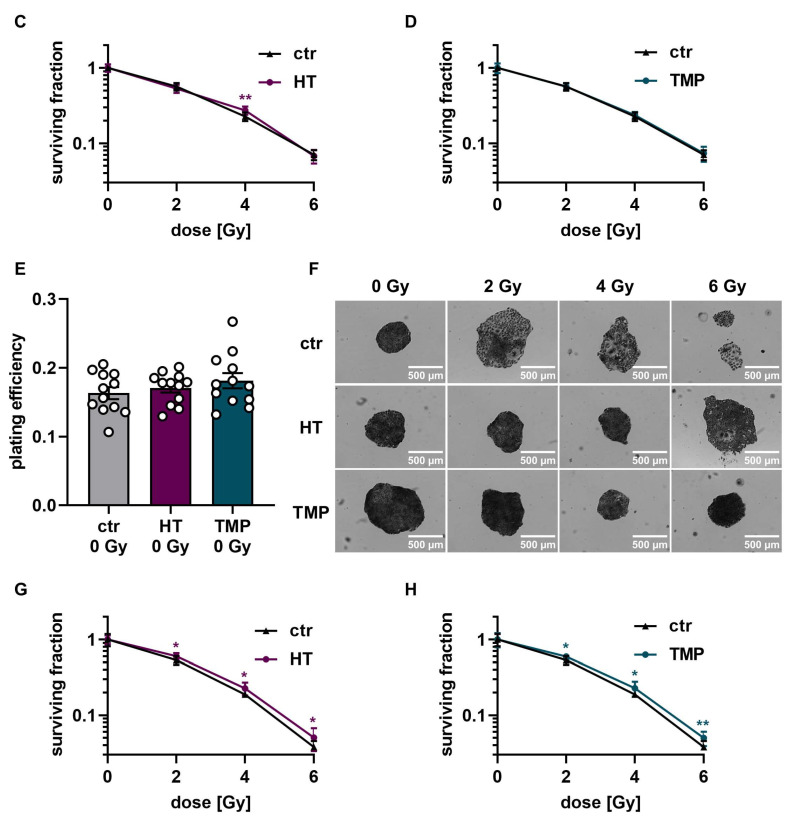
Colony formation assay of HaCaT cells after treatment with HT and TMP 24 h before and during irradiation, respectively. (**A**–**D**): Treatment with antioxidants 24 h before irradiation leads to a significantly higher plating efficiency compared to untreated controls at 0 Gy, indicating the higher proliferative potential of the treated cells (**A**). Untreated cells show changes in morphology after irradiation with 2, 4, and 6 Gy, while HT- and TMP-treated cells each maintain the original morphology with decreased post-irradiation changes (**B**). Incubation with HT results in slight but significant radioresistance for a dose of 4 Gy (**C**). TMP treatment has no effect on radioresistance (**D**). (**E**–**H**) show treatment with antioxidants during irradiation. The use of HT or TMP during irradiation has no substantial effect on the plating efficiency of HaCaT at 0 Gy (**E**). We observed the same changes in the morphology of control cells after irradiation, while antioxidant-treated cells largely preserved the original morphology (**F**). Both antioxidants enhance the radioresistance of cells. The effects increase with higher irradiation doses (**G**,**H**). All experiments were repeated at least three times in triplicate. *p* values < 0.05 were deemed significant (* *p* < 0.05; ** *p* < 0.01; error bars indicate the standard error of the mean (s.e.m.)).

**Figure 7:**
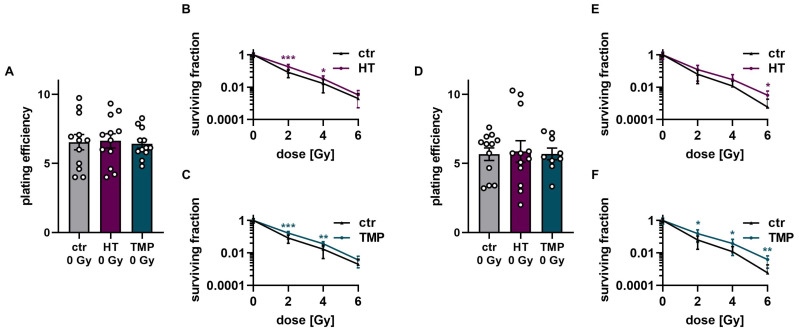
Colony formation assay of HUVECs after treatment with HT and TMP 24 h before and during irradiation, respectively. (**A**–**C**): Treatment with antioxidants 24 h before irradiation leads to no changes in plating efficiency compared to untreated controls at 0 Gy (**A**). Incubation with HT and TMP results in slight but significant radioresistance compared to similarly irradiated but otherwise untreated controls (**B**,**C**). (**D**–**F**): Treatment with antioxidants during irradiation. The use of HT or TMP during irradiation has no substantial effect on the plating efficiency of HUVECs at 0 Gy (**D**). Both antioxidants enhance the radioresistance of cells. The effects increase with higher irradiation doses (**E**,**F**). All experiments were repeated at least three times in triplicate. *p* values < 0.05 were deemed significant (* *p* < 0.05; ** *p* < 0.01; *** *p* < 0.001; error bars indicate the standard error of the mean (s.e.m.)).

To study HT- and TMP-induced changes in the effectiveness of ionizing radiation, we quantified clonogenic cell survival after radiation doses of 2 Gy, 4 Gy, and 6 Gy in HaCaT cells and HUVECs (Figure 6 and Figure 7). The treatment of HaCaT cells with each antioxidant 24 h before irradiation led to a significantly higher baseline cell survival level compared to untreated control cells (Figure 6A). However, only HT led to a slight increase in radioresistance in HaCaT cells at a dose of 4 Gy (Figure 6C). Treatment with HT or TMP during irradiation had no substantial effect on the plating efficiency in unirradiated cells. Nevertheless, we observed a significant radioprotective effect for both antioxidants, which increased with growing irradiation doses (Figure 6G,H). In both treatment regimens, we noticed a change in the appearance of untreated control cells after irradiation (Figure 6B,F). Our observations showed that the colonies of irradiated control cells contained fewer cells and grew in a less circular structure than the colonies of the non-irradiated cells. In addition, the irradiated cells had a significantly larger cytoplasm and larger nuclei compared to the non-irradiated cells. HT- or TMP-treated HaCaT cells largely maintained their original phenotype throughout all radiation doses. Only HT-treated cells showed slight changes in appearance after 6 Gy.

The treatment of HUVECs with HT and TMP both 24 h before and directly during irradiation had no effect on baseline cell survival (Figure 7A,D). However, significantly increased radioresistance of the cells was observed after treatment with HT and TMP for both treatment times (Figure 7B,C,E,F). In particular, HUVECs incubated with HT and TMP during irradiation showed increased radioresistance with increasing radiation doses (Figure 7E,F).

### 3.5. Antioxidants Promote Angiogenesis after Irradiation

We performed angiogenesis assays with HUVECs to determine antioxidant-induced changes in vascularization post-irradiation. Angiogenesis is crucial for wound healing [12], but the ability of endothelial cells to form vessels is compromised by irradiation [50].

**Figure 8:**
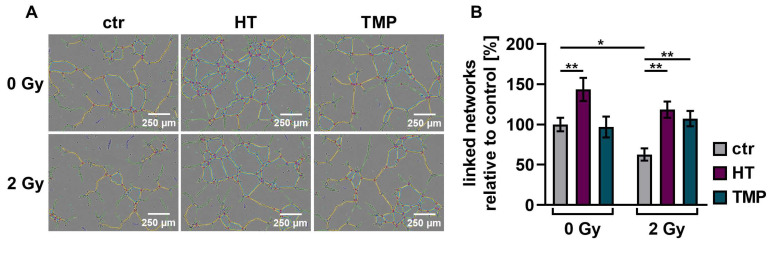

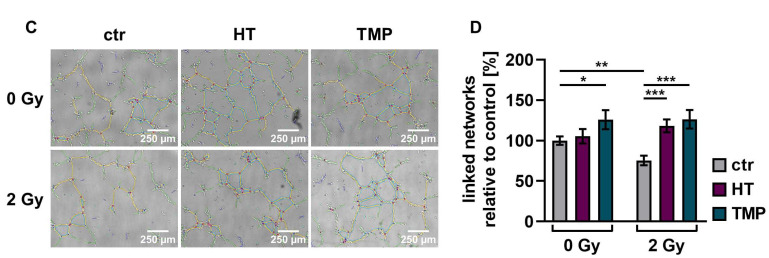
Angiogenic potential of HUVECs after radiation treatment. The angiogenesis of irradiated HUVECs was measured in cells treated with HT and TMP, respectively, 24 h before (**A**,**B**) and during irradiation (**C**,**D**) with 2 Gy. Single branches are shown in green, master junctions in red, master segments in yellow and vessel network is displayed in teal (**A**,**C**). Pictures for evaluation were taken 3 h after seeding and irradiation. Radiation treatment reduces the angiogenic potential of HUVECs. Angiogenesis was increased after the administration of antioxidants, regardless of the time of antioxidant treatment. All experiments were repeated at least three times in quintuplicate. *p* values < 0.05 were deemed significant (* *p* < 0.05; ** *p* < 0.01; *** *p* < 0.001; error bars indicate standard error of the mean (s.e.m.)).

We observed a significant decrease (about 30–40%) in the vascularization of the control HUVECs after irradiation with 2 Gy. The combination of irradiation and treatment with HT and TMP 24 h before irradiation caused significant increases in vessel formation of 88% (HT) and 71% (TMP) (Figure 8B). The administration of the respective antioxidants during irradiation also led to significant increases in angiogenesis (56% for HT and 67% for TMP-treated HUVECs; Figure 8D).

### 3.6. Enhanced Cell Migration and Proliferation with HT and TMP

Tissue regeneration plays an important role in wound closure after injury or surgery, and the proliferative and migrative capacities of fibroblasts and keratinocytes determine successful wound healing [51]. These processes are adversely affected by ionizing radiation [52].

**Figure 9:**
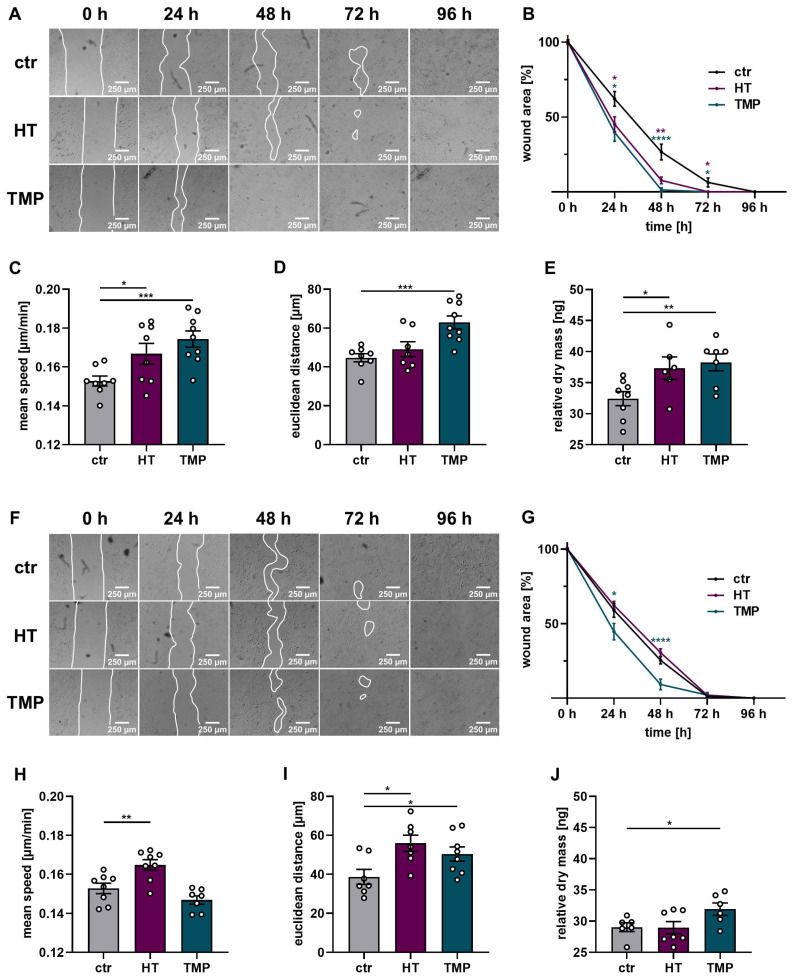
Wound healing, proliferation, and migration of irradiated HaCaT cells. Wound healing, migration, and dry mass measurements after the treatment of HaCaT with HT or TMP 24 h before (**A**–**E**) and during irradiation (**F**–**J**) with 2 Gy. (**A**,**B**) Wound closure after radiation treatment was accelerated in HT- and TMP-treated HaCaT cells with treatment 24 h before irradiation. (**C**,**E**) Cell speed, distance traveled over 24 h, and dry mass increment 24 h after irradiation in ng per field of view as a readout for proliferation were also increased. (**F**,**G**) Wound closure was also improved in cells treated with TMP immediately before irradiation. (**H**,**J**) Cell speed, distance traveled over 24 h, and dry mass increment 24 h after irradiation in ng per field of view also tended to be increased. All experiments were repeated at least three times in triplicate. *p* values < 0.05 were deemed significant (* *p* < 0.05; ** *p* < 0.01; *** *p* < 0.001, **** *p* < 0.0001; error bars indicate the standard error of the mean (s.e.m.)).

A wound healing test in the form of a scratch assay, as well as time-dependent migration and proliferation measurements via DHM with HaCaT, was performed to assess the protective effects of HT and TMP in this context. The results are shown in Figure 9.

Wound closure was significantly impaired in HaCaT cells irradiated with the standard dose of 2 Gy (time to wound closure 96 h). The administration of HT and TMP 24 h before irradiation substantially improved the ability of the cells to close the gaps in the cell layer (time to wound closure 72 h (HT) or 48 h (TMP)). Incubation with each antioxidant during irradiation only resulted in significantly faster wound closure for TMP treated cells (time to wound closure 72 h). These results were confirmed by time-dependent migration measurements via DHM for antioxidant treatment 24 h before irradiation. HaCaT cells treated with HT or TMP showed a significant increase in migration speed compared to control cells (Figure 9C) accompanied by an increase in total and Euclidian migration distance (Figure 9C,D). Treatment with HT or TMP during irradiation led to a significantly enhanced Euclidian distance over 24 h, and in the case of HT-treated HaCaT cells, to a significantly increased migration speed. Longitudinal DHM measurements showed a significantly higher cell dry mass 24 h after irradiation for HaCaT cells treated with HT or TMP 24 h before and during irradiation but only for TMP-treated cells with significance compared to control cells (Figure 9E,J).

## 4. Discussion

In this study, we found that that treatment with HT or TMP prior to and during irradiation show potential as radioprotective substances in normal skin cells, as they promote post-radiogenic migration, proliferation, angiogenesis, and clonogenic survival while leading to a reduction in ROS and attenuating inflammation.

### 4.1. Influence of HT and TMP on ROS, DNA Double-Strand Breaks, and Inflammation

Ionizing radiation-mediated intracellular processes depend on the formation of ROS [14]. ROS-associated cellular and molecular damage leads to the activation of inflammation-associated pathways, including NFκB, STAT3, and HIF-1 [53], as well as the secretion of inflammatory cytokines, such as IL-1, IL-6, IL-8, IL-33, TNF α, and TGF-β [54]. Pro-inflammatory cytokines and free radicals may then enter a positive feedback loop [55,56], promoting chronic inflammation [57,58] and attenuating wound healing [59]. Hence, impeding these processes in healthy tissue may reduce radiation-induced tissue toxicity. Intriguingly, we observed reductions in ROS and DNA double-strand breaks in irradiated HaCaT cells and HUVECs, as well as the downregulation of CXCL1, IL-6, IL-8, TIMP1, and TIMP2 after treatment with HT or TMP.

- CXCL1 is a pro-inflammatory chemokine, which is connected to fibrosis of the heart, lung, liver, and kidney tissue, as well as skin-related diseases (e.g., psoriasis, sunburn, itchy skin) [60,61].
- Initially, IL-6 and IL-8 have a positive function in acute wound healing by stimulating the proliferation and formation of antimicrobial peptides in keratinocytes. However, excessive secretion of these pro-inflammatory cytokines can lead to chronic inflammation and delayed wound healing [58]. Both interleukins are also involved in inflammatory reactions in endothelial cells following γ-ray irradiation [49]. The secretion of IL-8 is enhanced by oxidative stress, which leads to the formation of inflammatory cells and causes further increases in oxidative stress factors [62].
- Matrix metalloproteinases (MMPs) govern key wound-healing processes [63] and interact with tissue inhibitor of metalloproteinases (TIMPs). The dysregulation of this intricate interplay, as observed here, has been associated with chronic non-healing wounds [64].

In summary, we consistently find reduced ROS formation, as well as decreased DNA double-strand breaks and attenuated pro-inflammatory and anti-regenerative signaling. Our results are consistent with those of prior studies. The administration of HT has been linked to reduced DNA damage after UV irradiation [30] and the downregulation of IL-6 and IL-8 [65], as well as the dysregulation of the MMP/TIMP axis [17,20]. TMP treatment has been associated with reductions in ROS and IL-6 and IL-8 [23,26].

The partially differing results between HaCaT cells and HUVECs can be attributed to various biological factors. HaCaT cells are immortalized human keratinocytes derived from the epidermis of the skin. They possess specific mechanisms to cope with environmental stressors such as UV radiation and physical injury, which may influence their responses to different treatments. HUVECs are human umbilical vein endothelial cells derived from the inner lining of blood vessels. They play a central role in maintaining vascular homeostasis and function, which influences their sensitivity to stress and their repair mechanisms.

Metabolic differences, as well as different expression of receptors and enzymes of the two cell types, may lead to varying sensitivities to DNA damage and influence their ability to cope with oxidative stress and repair DNA damage.

### 4.2. Radioresistance

About two-thirds of radiation-induced DNA damage is due to ROS [66,67]. As a result of extensive DNA damage, cell death occurs [67,68]. Radioresistance describes the ability of cells to survive and grow despite irradiation-induced DNA damage [68]. While unwelcome in cancer cells, radioresistance in healthy tissue may decrease treatment-related side effects [69]. We found that treatment with HT and TMP mostly induced post-radiogenic clonogenic cell survival, indicative of enhanced radioresistance. The effect seemed to be more pronounced if antioxidant treatment occurred immediately before irradiation. However, there were some notable differences between our experiments, suggesting two hypotheses:

- Loss of antioxidative effects seemed to be more rapid in HaCaT cells than in HUVECs if treatment occurred 24 h before radiotherapy: In HaCaT cells, treatment 24 h before radiotherapy primarily induced changes independent of radiotherapy, like the increase in colony formation in unirradiated cells (Figure 6). Conversely, radiation-induced differences, like survival fractions (Figure 6), ROS formation (Figure 3), or cytokine measurements (Figure 5), were less prominent if treatment was performed 24 h before irradiation compared to treatment directly before RT.

Meanwhile, in HUVECs, antioxidant-induced effects remained prominent for radiation-induced changes even if treatment occurred 24 h before irradiation, including for survival fractions (Figure 7), ROS formation (Figure 3), and cytokine measurements (Figure 5).

Differences may be due to faster proliferation rates in HaCaT cells, where the doubling time is 28 h [70], while HUVEC cultures typically need a higher doubling time if not cultured in very early passages [71]. This may lead to the faster exhaustion of antioxidants in HaCaT cultures.

- However, some oxidative capacity remained for both cultures even if antioxidants were administered 24 h before irradiation: D’Angelo et al. found that HT was rapidly metabolized within 12–16 h, resulting in increased levels of the metabolite homovanillic alcohol [72]. Homovanillic alcohol, while less potent in its function than HT, retains some antioxidative properties [73]. These characteristics are likely responsible for the residual antioxidative effects observed after radiotherapy treatment in cells treated with HT 24 h before irradiation. Meanwhile, TMP is metabolized more slowly as Canesi and colleagues found that 70% of TMP was still present after 24 h at 37 °C [27].

This may be the reason why effects are antioxidant effects are strongest if antioxidants are present during irradiation, but some more limited effects remain if treatment is performed 24 h before irradiation.

In summary, our findings suggest that treatment with TMP and HT renders a sizable radioresistance effect, but the application of antioxidants should occur within a short timeframe before radiotherapy treatment.

### 4.3. Angiogenesis

Angiogenesis is an important factor for successful wound healing but may be attenuated by IR [6,11,50,52,74]. Therefore, postoperative radiotherapy is typically not started before some weeks of unimpeded post-surgical wound healing have passed [10]. Previous studies have found that HT and TMP may positively influence angiogenesis [18,26]. We show that both may substantially accelerate post-radiogenic angiogenesis, again supporting the hypothesis that antioxidants may aid wound healing after radiotherapy. Notably, HT was pro-angiogenic at low doses (within the range that we applied), but it has previously been described as anti-angiogenic at higher concentrations [18]. Hence, HT dose may not be increased without paying specific attention to this in future studies.

### 4.4. Proliferation, Migration and Wound Healing

As an essential component of wound healing, epithelialization is considered a crucial parameter for its success [63]. Keratinocytes at the wound edge start to migrate, while the trailing keratinocytes start proliferating to ensure proper wound closure [75]. We found that HT and TMP promote wound closure in HaCaT cells after irradiation. In addition, DHM measurements showed increased migration speed and distance in HT- and TMP-treated HaCaT cells, as well as accelerated proliferation after antioxidant treatment. This approach may support enhanced wound closure.

Some limitations need to be considered when interpreting this study. First, while we consistently tested two diverse cell cultures, healthy tissue is more heterogeneous and individual patterns need further study. However, effects were largely homogeneous between HUVECs and HaCaT cells. Second, definitions of colonies may vary as some standardized protocols suggest a minimum of 50 cells [76], while others use a minimum of 25 cells as a cutoff, like our investigation [77,78,79,80]. Finally, future in vivo studies are needed to the confirm favorable effects of antioxidant treatment. Notably, the six-well plates that we used for irradiation may not represent the physiologically present oxygen tension after irradiation in vivo.

For in vivo application, we envision the transdermal application of antioxidants. This would allow for the selective administration of agents to radiation-exposed healthy tissue (e.g., the skin) while limiting exposure of tumor cells (e.g., from breast cancer) to radioresistance-inducing substances. Conversely, intravenous or oral application of antioxidants may disproportionately enhance tumor radioresistance, considering that hypervascularization of tumors may increase tumor cell exposure to antioxidants. Studies in human skin models have shown appreciable transdermal absorption of HT that was even more strongly increased in inflamed skin [81], a condition likely present in radiotherapy-exposed dermis. Studies not including radiotherapy have already demonstrated the anti-inflammatory effects of transdermal HT application in an ex vivo model [82].

## 5. Conclusions

Side effects during radiotherapy remain a common problem and negatively affect patients’ quality of life. Our work has shown that treatment with HT or TMP has radio-protective effects on both human keratinocytes and human primary endothelial cells.

The findings point to a possible reduction in radiation-induced normal tissue toxicity after treatment with antioxidants, suggesting therapeutic potential and underlining the need for further study. Future investigations should include in vivo or complex in vitro tissue modeling to assess the potential applications of these antioxidants in a physiological setting.

## Figures and Tables

**Table 1:** Overview of gene expression results. Shown below are fold changes relative to ctr 2 Gy for different genes of interest. All experiments were repeated at least three times in triplicate. Student’s *t*-test was performed, HT and TMP 2 Gy were compared to ctr 2 Gy, and *p* values < 0.05 were deemed to be significant (* *p* < 0.05; ** *p* < 0.01; **** *p* < 0.0001; errors indicate the standard error of the mean (s.e.m.)).

Cell Type	Time of Treatment	2 Gy	Gene of Interest				
			CXCL1	IL-6	IL-8	TIMP1	TIMP2
**HaCaT**	**24 h before IR**	**ctr**	1.00	1.00	1.00	1.00	1.00
		**HT**	0.88 ± 0.06 *	0.74 ± 0.08 **	0.90 ± 0.08	0.85 ± 0.09	0.87 ± 0.06 *
		**TMP**	1.22 ± 0.09 *	0.83 ± 0.08 *	1.40 ± 0.08 ****	0.87 ± 0.08 *	0.77 ± 0.08 **
	**During IR**	**ctr**	1.00	1.00	1.00	1.00	1.00
		**HT**	0.81 ± 0.08 *	0.85 ± 0.08 *	0.81 ± 0.11 *	0.78 ± 0.07 **	0.91 ± 0.15
		**TMP**	0.92 ± 0.08	0.86 ± 0.05 **	0.96 ± 0.21	1.16 ± 0.09	0.94 ± 0.10
**HUVECs**	**24 h before IR**	**ctr**	1.00	1.00	1.00	1.00	1.00
		**HT**	0.94 ± 0.11	0.80 ± 0.14	0.90 ± 0.10	0.92 ± 0.04 *	0.87 ± 0.06 *
		**TMP**	0.87 ± 0.08	0.65 ± 0.16 *	0.95 ± 0.05	0.92 ± 0.06	0.88 ± 0.07
	**During IR**	**ctr**	1.00	1.00	1.00	1.00	1.00
		**HT**	0.88 ± 0.08	0.87 ± 0.11	0.79 ± 0.14	0.79 ± 0.18	1.04 ± 0.05
		**TMP**	0.82 ± 0.10	0.64 ± 0.13 **	1.08 ± 0.14	0.80 ± 0.18	0.96 ± 0.12

## Data Availability

Data are contained within this article.

## Supplementary Materials

The following supporting information can be downloaded at https://www.mdpi.com/article/10.3390/antiox13080961/s1, Figure S1: Cytokine array to quantify the radiation-induced release of pro-inflammatory cytokines. Shown here are the results of the unirradiated HUVEC treated with HT and TMP in the same way as the irradiated cells. A Cytokine array was performed with supernatants from HUVECs (A) treated with HT or TMP 24 h before (B) and during (C) irradiation with 2 Gy (*n* = 1). The supernatants were collected 48 h after irradiation. Chemiluminescence was used to measure and quantify cytokine binding to the membrane. Intensity was evaluated with the help of ImageJ software. Eighty different cytokines were tested, and cytokines with relevant changes are shown in (B,C); Table S1: TaqMan probes used for qRT-PCR. Probes were purchased from Thermo Fisher Scientific; Table S2: Components of Loeffler staining solution; Table S3: Overview of gene expression results for untreated cells. Shown below are fold changes relative to ctr 0° Gy for different genes of interest. All experiments were repeated at least three times in triplicate. Student’s *t*-test was performed, ctr 2° Gy was compared to ctr 0° Gy, and *p* values < 0.05 were deemed significant (* *p* < 0.05; ** *p* < 0.01; *** *p* < 0.001; **** *p* < 0.0001; errors indicate standard error of the mean (s.e.m.)).
