# Supplementary material for: Antioxidants Hydroxytyrosol and Thioredoxin-Mimetic Peptide CB3 Protect Irradiated Normal Tissue Cells
Source: Antioxidants (Basel). 2024 Aug 7;13(8):961. doi: 10.3390/antiox13080961 (PMC11351936; doi:10.3390/antiox13080961)
Supplement: Supplementary file 1 [file antioxidants-13-00961-s001.zip › antioxidants-3097751-supplementary.pdf]

Supplementary material

Supplementary table S1: TaqMan probes used for qRT-PCR. Probes were purchased from Thermo Fisher Scientific.

RNA	Gene-ID
18S	HS99999901_S1
CXCL1	Hs00236937_m1
IL-6	Hs00174131_m1
IL-8	Hs00174103_m1
TIMP1	HS01092512_g1
TIMP2	Hs00234278_m1

Supplementary table S2: Components of Loeffler staining solution.

	Component	Amount
**Stock solution**	Methylen blue	2 g
	Ethanol 70%	100 mL
**Staining solution**	Stock solution	30 mL
	Aqua dest.	69 mL
	NaOH 1%	1 mL

Supplementary table S3: **Overview of gene expression results for untreated cells.** Shown below are fold changes relative to ctr 0 Gy for different genes of interest. All experiments were repeated at least three times in triplicates. Students t-test was performed, ctr 2 Gy were compared to ctr 0 Gy, p values <0.05 were deemed significant (*p < 0.05; **p < 0.01; ***p < 0.001, ****p < 0.0001, errors indicate standard error of the mean (s.e.m.)).

Cell type	Timepoint of treatment	ctr	Gene of interest				
			CXCL1	IL-6	IL-8	TIMP1	TIMP2
HaCaT	24 h before IR	0 Gy	1	1	1	1	1
		2 Gy	1,11 ± 0,06	1,26 ± 0,14*	1,04 ± 0,05	1,23 ± 0,09**	1,43 ± 0,18*
	during IR	0 Gy	1	1	1	1	1
		2 Gy	1,28 ± 0,07***	2,56 ± 0,32****	0,96 ± 0,09	1,01 ± 0,12	1,60 ± 0,17**
HUVEC	24 h before IR	0 Gy	1	1	1	1	1
		2 Gy	2,03 ± 0,30**	1,27 ± 0,12	2,32 ± 0,34***	1,30 ± 0,14*	1,32 ± 0,11**
	during IR	0 Gy	1	1	1	1	1
		2 Gy	1,43 ± 0,14**	1,67 ± 0,21**	1,21 ± 0,10*	1,09 ± 0,09	1,39 ± 0,11**

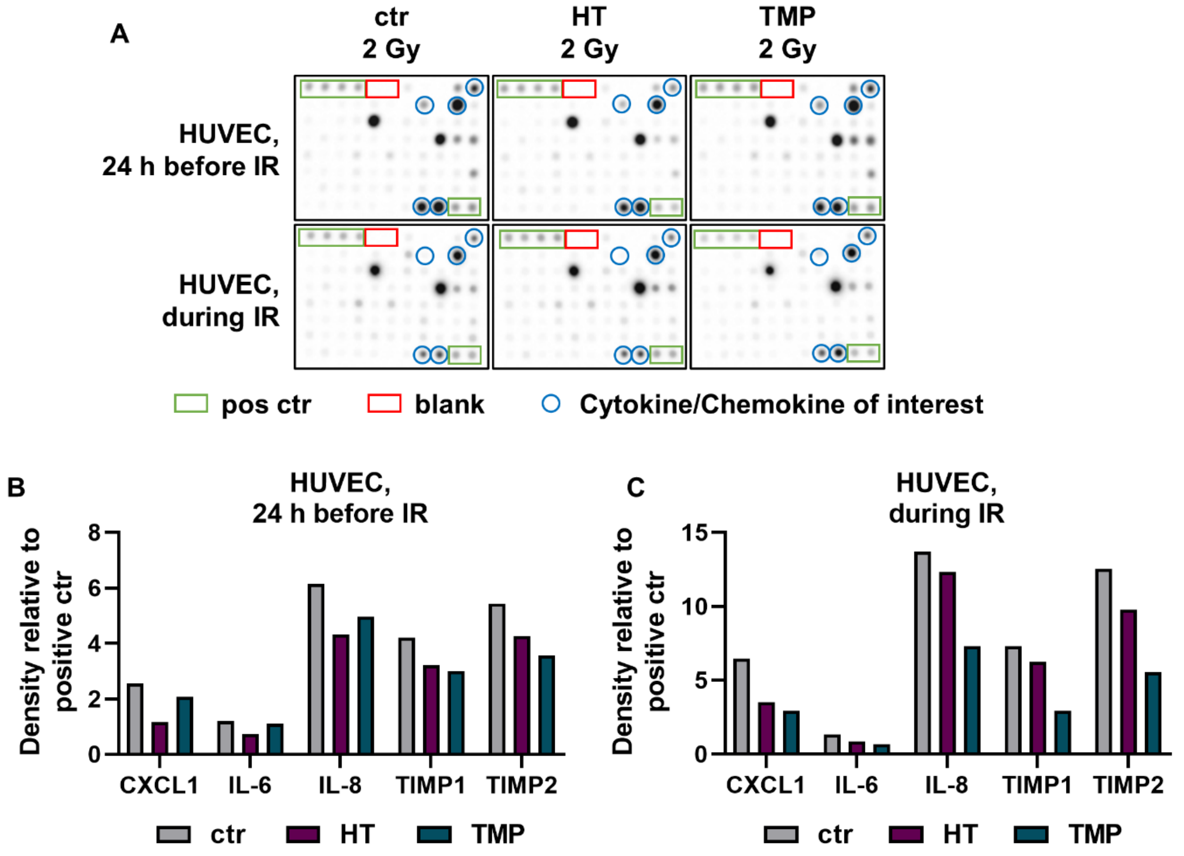

Supplementary figure S1. Cytokine array to quantify radiation-induced release of pro-inflammatory cytokines. Shown here are the results of the unirradiated HUVEC treated with HT and TMP in the same way as the irradiated cells. A Cytokine array was performed with supernatants from HUVECs (**A**) treated with HT or TMP 24 h before (**B**) and during (**C**) irradiation with 2 Gy (n = 1). The supernatants were collected 48 h after irradiation. Chemiluminescence was used to measure and quantify cytokine binding to the membrane. Intensity was evaluated with the help of ImageJ software. 80 different cytokines were tested and cytokines with relevant changes were shown in **B** and **C**.
